# Demographic disparities in receipt of care at a comprehensive cancer center

**DOI:** 10.1002/cam4.5992

**Published:** 2023-04-28

**Authors:** Kedar Kirtane, Yayi Zhao, Rossybelle P. Amorrortu, Lindsay N. Fuzzell, Susan T. Vadaparampil, Dana E. Rollison

**Affiliations:** ^1^ Department of Head and Neck‐Endocrine Oncology Moffitt Cancer Center Tampa Florida USA; ^2^ Office of Community Outreach Engagement, and Equity, Moffitt Cancer Center Tampa Florida USA; ^3^ Department of Cancer Epidemiology Moffitt Cancer Center Tampa Florida USA; ^4^ Department of Health Outcomes & Behavior Moffitt Cancer Center Tampa Florida USA

**Keywords:** cancer care, epidemiology, healthcare disparities, National Cancer Institute cancer center, patient care/delivery of health care

## Abstract

**Background:**

National Cancer Institute cancer centers (NCICCs) provide specialized cancer care including precision oncology and clinical treatment trials. While these centers can offer novel therapeutic options, less is known about when patients access these centers or at what timepoint in their disease course they receive specialized care. This is especially important since precision diagnostics and receipt of the optimal therapy upfront can impact patient outcomes and previous research suggests that access to these centers may vary by demographic characteristics. Here, we examine the timing of patients' presentation at Moffitt Cancer Center (MCC) relative to their initial diagnosis across several demographic characteristics.

**Methods:**

A retrospective cohort study was conducted among patients who presented to MCC with breast, colon, lung, melanoma, and prostate cancers between December 2008 and April 2020. Patient demographic and clinical characteristics were obtained from the Moffitt Cancer Registry. The association between patient characteristics and the timing of patient presentation to MCC relative to the patient's cancer diagnosis was examined using logistic regression.

**Results:**

Black patients (median days = 510) had a longer time between diagnosis and presentation to MCC compared to Whites (median days = 368). Black patients were also more likely to have received their initial cancer care outside of MCC compared to White patients (odds ratio [OR] and 95% confidence interval [CI] = 1.45 [1.32–1.60]). Furthermore, Hispanics were more likely to present to MCC at an advanced stage compared to non‐Hispanic patients (OR [95% CI] = 1.28 [1.05–1.55]).

**Conclusions:**

We observed racial and ethnic differences in timing of receipt of care at MCC. Future studies should aim to identify contributing factors for the development of novel mitigation strategies and assess whether timing differences in referral to an NCICC correlate with long‐term patient outcomes.

## Graphical abstract

There are racial differences in timing of receipt of care at our NCI cancer center. Black patients were more likely to have initial care outside of our center, while Hispanic patients were more likely to present with advanced stage.

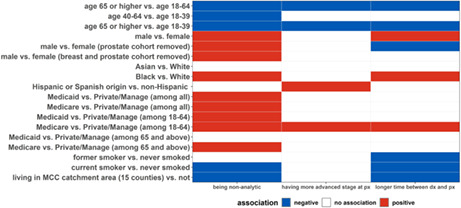

## 1 INTRODUCTION

There are over 70 National Cancer Institute (NCI)‐designated cancer centers in the United States. These designations are awarded to institutes deemed by the NCI to meet rigorous standards for multidisciplinary cancer care.^1^ These centers are at the forefront of cancer care, and patients who seek care at these institutions may have access to cutting‐edge treatments and therapies that may not be available elsewhere. Recent data has demonstrated improved outcomes for NCI cancer centers (NCICC) relative to non‐NCI centers.^2^ For example, one study found that NCICC had lower adjusted surgical mortality rates than control hospitals for certain surgical procedures.^2^ Other studies report superior overall survival for patients with newly‐diagnosed cancers at NCICCs versus non‐designated centers.^3^, ^4^ Access to clinical trials may contribute to this survival benefit.

In addition to access to clinical trials, data suggest that patients may receive care more guideline‐based care at NCICCs compared to community settings,^5^, ^6^, ^7^ especially in cancers providing multidisciplinary care.^5^ One study using the Texas Cancer Registry found that care at a NCICC correlated with a higher rate of guideline‐based care and a 39% relative risk reduction in mortality at 2 years from initial diagnosis.^5^ Another study using the Survival, Epidemiology, and End Results (SEER)‐Medicare database demonstrated improvements in long‐term survival for patients undergoing surgery for colorectal cancer at NCICCs compared to non‐designated centers.^6^ Other studies involving patients with genitourinary cancers,^7^ acute myeloid leukemia,^8^ breast or lung cancers,^9^ and ovarian cancer^10^ have observed improvements in survival/mortality and fewer postoperative complications among patients treated at NCICCs compared to those patients treated at non‐NCICCs. Overall, the evidence suggests that patients treated at NCICC tend to have better outcomes,^5^, ^6^, ^7^, ^8^, ^9^, ^10^ although the degree of difference may vary depending on the type of cancer and other patient and treatment factors.

Relatively less is known about patient or disease characteristics associated with the timing of care at these NCICCs. This is especially true for underrepresented minorities for whom disparities in prevention and treatment remain stark.^11^ Furthermore, among patients who are ultimately treated at an NCICC, variation exists in when they receive care relative to their disease course. This is critical, as early care at an NCICC may be important for outcome.^3^ One population‐based study demonstrated worse overall survival for cancer patients not receiving first course treatment at an NCICC.^3^ Possible reasons for improved outcomes associated with earlier care at NCICCs include more accurate cancer diagnoses, conducting next‐generation sequencing when appropriate to determine relevant treatments, and offering novel clinical trials for patients with a paucity of standard treatment options. Importantly, patients who seek their initial treatment outside of an NCICC may undergo lines of systemic therapy that render them ineligible for clinical trials. Furthermore while more recent data suggests there may be delays in time to treatment initiation at academic centers for certain cancer types, it is unclear whether there are specific patient characteristics associated with such delays.^12^

To evaluate possible disparities in early care at NCICCs, we sought to determine patient characteristics associated with receipt of care at our NCICC. Specifically, we examined the timing of patients' presentation to our center relative to their initial diagnosis across several demographic characteristics among patients with cancers.

## 2 MATERIALS AND METHODS

### 2.1 Study design and population

We conducted a retrospective study among Moffitt Cancer Center (MCC) patients to examine the association between patient characteristics and the timing of receipt of care at our NCICC relative to the patient's cancer diagnosis. Patients were included in the study if they (1) were diagnosed at aged ≥18 with any of the five screening‐detectable cancers that reflect the largest burden in the MCC catchment area (breast cancer, colon cancer, lung cancer, melanoma, or prostate cancer), (2) presented for the first time at MCC between December 2008 and April 2020, and (3) received a diagnosis and/or active treatment at MCC. The final sample size included 41,539 patients comprising those with breast cancer (*n* = 12,334), colon cancer (*n* = 3086), lung cancer (*n* = 10,604), melanoma (*n* = 8821), and prostate cancer (*n* = 6694). The study adheres to the tenets of the Declaration of Helsinki and was reviewed by the Advarra Institutional Review Board and determined to be exempt, Pro00044703.

### 2.2 Data collection

Patient demographic and clinical characteristics were obtained from the Cancer Registry including age at diagnosis, gender, race, ethnicity, county of residence at initial presentation to MCC, payment method, class of case, cigarette smoking history, date of diagnosis, tumor stage at diagnosis, date of first presentation to MCC, and tumor stage at presentation. Cancer stages according to Surveillance, Epidemiology, and End Results (SEER) summary staging system were selected for the current analysis since it was available at both the time of diagnosis and presentation to MCC. Patients were categorized as living in the 15‐county MCC catchment area (2016–2021) at the time of presentation.

### 2.3 Measurements of the timing of receipt of care

Several approaches were taken to define groups of patients whose receipt of care at MCC could be considered “delayed”. First, the timing of presentation to Moffitt relative to the disease course was considered by assessing patients' class of case, as recorded in the Cancer Registry, including analytic and non‐analytic cases. Analytic cases were defined as a case in which the patient is diagnosed and/or receiving first course treatment at MCC (*n* = 32,608). Non‐analytic cases were defined as a case in which the patient's first contact at MCC follows prior treatment at a different facility (*n* = 8931). The stage at cancer diagnosis relative to the stage at presentation to Moffitt was considered, and the subgroup of patients whose tumor stage at presentation was more advanced than their original stage at diagnosis was identified and compared to those who had stage concordant at presentation. Finally, within non‐analytics, length of time between date of diagnosis and date of presentation was evaluated.

### 2.4 Statistical analysis

Demographic and clinical factors were cross tabulated by class of case, and associations between patient factors and class of case were estimated using logistic regression adjusted for cancer cohort. A multivariable logistic regression model was constructed to include cancer type and all factors that were significantly associated with class of case from the cohort‐adjusted analysis. A backward elimination process was performed on the multivariable model to drop the factor with the largest *p*‐value at each run until all factors remaining had *p*‐values ˂0.05. Since Medicare is generally only available to patients aged ≥65, an interaction term for age and payment method was included in the multivariable model.

Separately for analytic and non‐analytic cases, the stage at presentation was plotted against the stage at diagnosis to identify four patient groups: (1) those whose tumor stage at presentation to MCC was the same as their tumor stage at initial diagnosis (concordant stage at presentation) (2) those whose tumor stage at presentation was more advanced than that at diagnosis, (3) those whose tumor stage at diagnosis was more advanced than that at presentation, and (4) those whose stage data were missing at either time point. Stage distributions were also stratified by types of cohort‐defining cancer. Of note, the tumor stage at diagnosis could have been more advanced than the stage at presentation to Moffitt if the patient received treatment elsewhere (prior to their presentation at Moffitt), resulting in a tumor stage that was less advanced at presentation than the original stage at diagnosis.

The characteristics of patients with more advanced stage at presentation were compared to the non‐analytic patients who had concordant stage at presentation. Cohort‐adjusted logistic regression was used to estimate the association between each characteristic and whether a patient had a more advanced stage at presentation. Subsequently, a backward elimination process was performed on a multivariable logistic regression model including cancer type and all factors that were significantly associated with a more advanced stage at presentation. An interaction term for age and payment method was also included in the model. In addition, the univariable logistic regression models were stratified by cancer type.

The time between initial cancer diagnosis and presentation at MCC was also analyzed among non‐analytic cases. The median and interquartile range of the number of days between the date of cancer diagnosis and the date of presentation were summarized for each group. Wilcoxon rank‐sum and Kruskal–Wallis test were used to compare the lag time as appropriate. To further explore disparities in the time between initial cancer diagnosis and presentation to MCC, analyses were conducted examining two sub‐intervals: (1) time between cancer diagnosis and initiation of first course treatment and (2) time between the end of first course treatment and presentation to MCC. Patients were included in this exploratory analysis if they received any of the following treatment modalities as a part of their first course treatment outside of Moffitt: chemotherapy, hormone therapy, immunotherapy, radiation, surgery, and organ transplantation. Time between cancer diagnosis and initiation of first course treatment was compared across patient groups as defined by ethnicity using Wilcoxon rank‐sum test and by race using Kruskal–Wallis test, followed by post hoc analysis with adjustment for multiple comparisons. The same methods were used to compare time between the end of first course treatment and presentation to MCC. All analysis were performed using R version 4.0.2 (RRID: SCR_001905).

Lastly, a heatmap‐like plot was generated to summarize the associations between patient characteristics and each of the three proxy indicators of delayed receipt of care described previously.

## 3 RESULTS

### 3.1 Class of case

After adjusting for cancer type, patients ≥65 at time of cancer diagnosis were less likely to be non‐analytic cases (odds ratio [OR] = 0.82; 95% confidence interval [CI] = 0.78–0.86) as compared with patients diagnosed before age 65 (Table 1). Further, an inverse trend was observed between age and non‐analytic status when the age groups were further divided into 18–39, 40–64, and ≥65. Males were more likely to be non‐analytic cases as compared with females (OR [95% CI] = 1.19 [1.11–1.26]). Black patients had significantly higher odds of being non‐analytic cases as compared with White patients (OR [95% CI] = 1.45 [1.32–1.60]) while Asian patients, and those of other race or multiple races did not differ significantly from White patients. Compared to patients with private/managed insurance, patients who self‐paid (OR [95% CI] = 0.80 [0.67–0.95]) or were insured through Tricare/Veteran's Affairs (OR [95% CI] = 0.83 [0.68–1.00]) were less likely to be non‐analytic whereas patients with Medicaid (OR [95% CI] = 1.30 [1.10–1.52]) or Medicare (OR [95% CI] = 1.10 [1.02–1.18]) were more likely to be non‐analytic cases (Table 1). Interestingly, among patients aged 18–64, Medicare holders were 77% more likely to be non‐analytic as compared to private/managed insurance holders, while the same association was weaker among patients aged ≥65 (OR [95% CI] = 1.25 [1.05–1.51]). Additionally, among patients <65, those with Medicaid (OR [95% CI] = 1.28 [1.08–1.51]) were more likely to be non‐analytic as compared with those with private/managed insurance, whereas among patients aged ≥65 Medicaid users were less likely to be non‐analytic (OR [95% CI] = 0.78 [0.30–1.70]). Current smokers (OR [95% CI] = 0.96 [0.90–1.01]) and former smokers (OR [95% CI] = 0.82 [0.75–0.90]) were less likely to be non‐analytic as compared to those who never smoked. Patients who resided in the MCC 15‐county catchment area (OR [95% CI] = 0.47 [0.44–0.50]) were less likely to be non‐analytic cases as compared with those who resided outside the catchment area. The final multivariable model retained age, gender, race, payment method, 15‐county catchment area, and the interaction between age and payment (Table 1). Of note, the proportions of non‐analytic patients varied by cancer type (breast = 15%, colon = 39%, lung =26%, melanoma = 14%, prostate = 29%).

**TABLE 1:** Association between patient characteristics and analytic case status among Moffitt Cancer Center patients (MCC) diagnosed with breast cancer, colon cancer, lung cancer, melanoma, and prostate cancer in 2008–2020.

Baseline characteristics	Class of case			
	Analytic cases	Non‐analytic cases	Odds ratio (95% confidence interval)	
			Cohort‐adjusted^a^	Multivariable
Age				
18–64	16,453 (50.5)	4714 (52.8)	1.00 (reference)	1.00 (reference)
65 or higher	16,155 (49.5)	4217 (47.2)	0.82 (0.78–0.86)	0.81 (0.67–0.97)
Age				
18–39	1505 (4.6)	390 (4.4)	1.00 (reference)	Not included^b^
40–64	14,948 (45.8)	4324 (48.4)	0.85 (0.75–0.96)	
65 or higher	16,155 (49.5)	4217 (47.2)	0.70 (0.62–0.79)	
Gender				
Female	18,522 (56.8)	4113 (46.1)	1.00 (reference)	1.00 (reference)
Male	14,084 (43.2)	4817 (53.9)	1.19 (1.11–1.26)	1.21 (1.11–1.32)
Race				
White	29,433 (90.6)	7835 (88.1)	1.00 (reference)	1.00 (reference)
Asian	447 (1.4)	124 (1.4)	1.06 (0.86–1.30)	0.98 (0.72–1.30)
Black	1608 (4.9)	668 (7.5)	1.45 (1.32–1.60)	1.26 (1.09–1.44)
Other/multiple	1011 (3.1)	264 (3.0)	0.93 (0.81–1.07)	0.83 (0.67–1.02)
Ethnicity				
Non‐Hispanic	30,288 (93.1)	8241 (92.9)	1.00 (reference)	Not included
Hispanic or Spanish origin	2237 (6.9)	632 (7.1)	1.03 (0.94–1.13)	
Payment method (all ages)				
Private/Manage	13,029 (40.4)	1562 (36.2)	1.00 (reference)	1.00 (reference)
Self‐pay/not insured	1533 (4.8)	150 (3.5)	0.80 (0.67–0.95)	0.77 (0.63–0.92)
Medicaid	1238 (3.8)	195 (4.5)	1.30 (1.10–1.52)	1.34 (1.13–1.59)
Medicare	15,331 (47.5)	2288 (53.0)	1.10 (1.02–1.18)	1.78 (1.54–2.05)
Tricare/VA	1118 (3.5)	122 (2.8)	0.83 (0.68–1.00)	0.80 (0.61–1.03)
Payment method (age 18–64)				
Private/Manage	11,737 (72.3)	1417 (66.8)	1.00 (reference)	Reference for interaction term
Self‐pay/not insured	1429 (8.8)	138 (6.5)	0.78 (0.64–0.93)	
Medicaid	1167 (7.2)	189 (8.9)	1.28 (1.08–1.51)	
Medicare	1216 (7.5)	305 (14.4)	1.77 (1.54–2.03)	
Tricare/VA	682 (4.2)	72 (3.4)	0.85 (0.66–1.09)	
Payment method (age 65 and above)				Interaction effect
Private/Manage	1292 (8.1)	145 (6.6)	1.00 (reference)	(age: payment)
Self‐pay/not insured	104 (0.6)	12 (0.5)	0.98 (0.50–1.78)	1.15 (0.57–2.16)
Medicaid	71 (0.4)	6 (0.3)	0.78 (0.30–1.70)	0.55 (0.19–1.31)
Medicare	14,115 (88.1)	1983 (90.3)	1.25 (1.05–1.51)	0.72 (0.57–0.91)
Tricare/VA	436 (2.7)	50 (2.3)	0.93 (0.65–1.30)	1.19 (0.77–1.84)
Cigarettes use				
Never	12,172 (43.5)	3332 (41.2)	1.00 (reference)	Dropped due to lack of significance
Former	12,217 (43.7)	3791 (46.9)	0.96 (0.90–1.01)	
Current	3594 (12.8)	967 (12.0)	0.82 (0.75–0.90)	
Whether address at diagnosis is within MCC catchment area (15 counties in FL^c^)				
No	6447 (19.9)	1653 (34.1)	1.00 (reference)	1.00 (reference)
Yes	26,024 (80.1)	3188 (65.9)	0.47 (0.44–0.50)	0.48 (0.45–0.52)
Cohort				
Breast	10,528 (32.3)	1806 (20.2)	Not applicable	1.00 (reference)
Colon	1873 (5.7)	1213 (13.6)		3.04 (2.67–3.46)
Lung	7853 (24.1)	2751 (30.8)		2.17 (1.96–2.40)
Melanoma	7611 (23.3)	1210 (13.5)		0.80 (0.70–0.90)
Prostate	4743 (14.5)	1951 (21.8)		1.77 (1.55–2.02)

^a^ The estimated odds ratio and confidence interval were calculated using logistic regression adjusting for cancer types the patient diagnosed with.

^b^ The three‐group age variable was not included in the multivariable analysis due to (1) not being significant in the univariable analysis, and (2) not being compatible with the binary age categorization used in the analysis of interaction between age and payment method.

^c^ The 15‐county MCC catchment area included the following counties in Florida: Charlotte, Citrus, Desoto, Hardee, Hernando, Highlands, Hillsborough, Lake, Lee, Manatee, Pasco, Pinellas, Polk, Sarasota, or Sumter.

**FIGURE 1:**
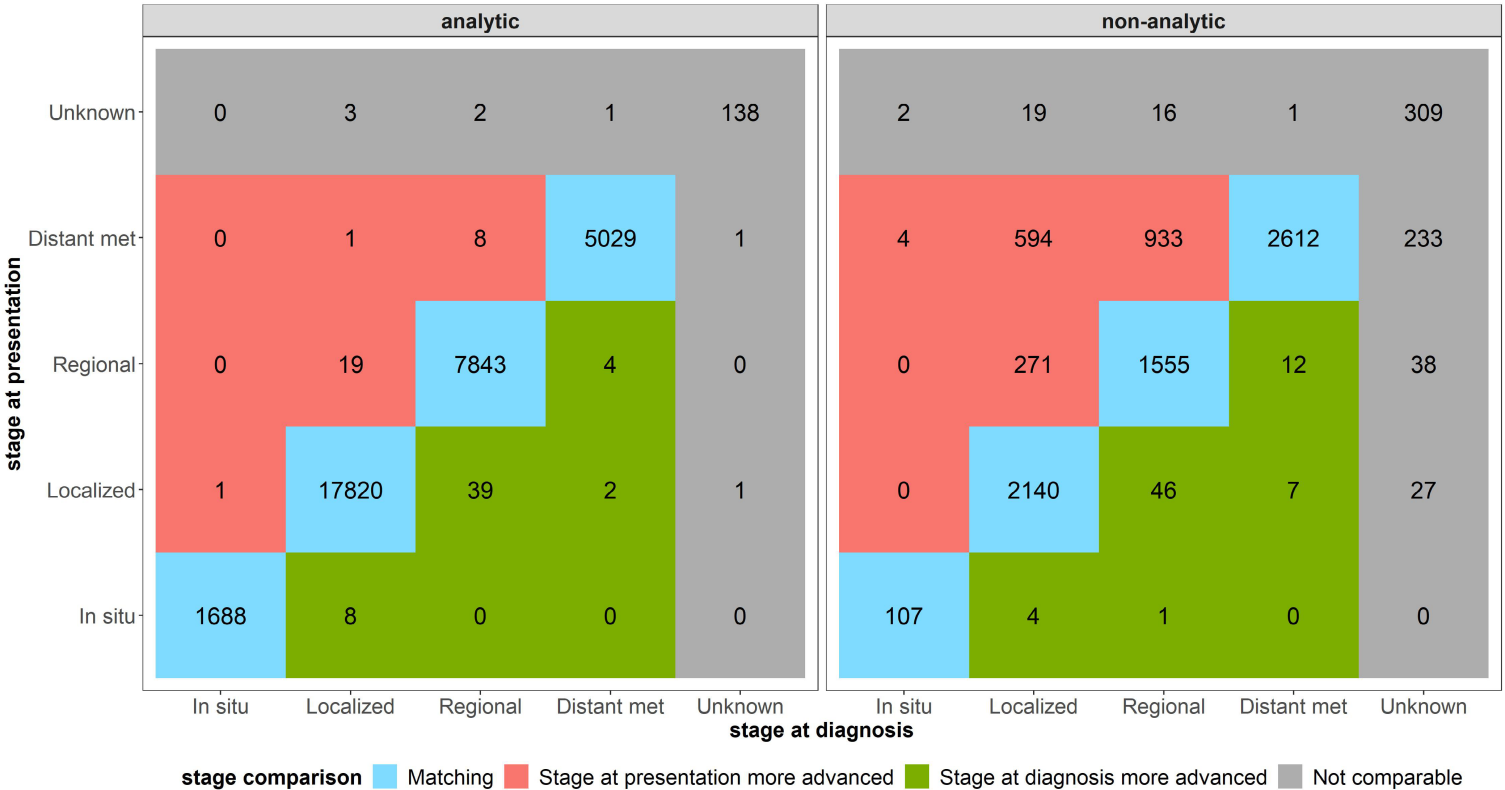
The blue color indicates patients whose stage at presentation matched with the stage at diagnosis, whereas the red color indicates patients whose stage at presentation was more advanced than their stage at diagnosis. Most of the patients whose stage at presentation was more advanced were non‐analytic patients which is reflective of the definition of non‐analytic patients as being those who have their first course therapy completed before coming to MCC. Thus, the non‐analytic status indicates delayed access to care at Moffitt (MCC), while the patients highlighted in red may represent a subset of extreme cases whose delayed access to care resulted in a more advanced stage when they presented at MCC.

Among analytic cases, 99% of the patients had the same tumor stage at diagnosis and at presentation while few patients had tumors that progressed to a more advanced stage at presentation as compared to their stage at diagnosis (Figure 1). However, only 72% of the non‐analytic cases had tumors that remained at the same stage at presentation, whereas 20% of non‐analytic cases had tumors that progressed to a more advanced stage from the time of diagnosis to the time of presentation (Figure 1). Among the 1802 non‐analytic cases whose tumor progressed between the two time points, 271 (15%) of the patients' tumors progressed from localized to regional, 594 (33%) progressed from localized to distant metastasis, while more than half (*n* = 933, 52%) progressed from regional to distant metastasis. A similar pattern was observed when stratifying the stage distributions by cancer type, with most analytic patients having stage concordant at presentation regardless of cancer type (Appendix A).

### 3.2 Stage at cancer diagnosis relative to the stage at presentation to Moffitt

Among non‐analytic cases, we examined the association between patient characteristics and whether the patient had a more advanced stage at presentation while adjusting for cancer type (Table 2). Patients aged ≥65 at diagnosis (OR [95% CI] = 0.85 [0.76–0.95]) were less likely to have a more advanced stage at presentation as compared to those who were younger. When dividing patients into age groups, we observed an inverse trend between age and the likelihood of having a more advanced stage at presentation. Race was not significantly associated with a more advanced stage at presentation while Hispanic patients were more likely to have a more advanced stage at presentation as compared with non‐Hispanic patients (OR [95% CI] = 1.28 [1.05–1.55]). Particularly among patients aged 18–64 at diagnosis, those with Medicare (OR [95% CI] = 2.17 [1.49–3.11]) were twice as likely to have a more advanced stage at presentation as compared with those with private/managed insurance. While not statistically significant, patients residing in the 15‐county catchment area were less likely to have a more advanced stage at presentation (OR [95% CI] = 0.86 [0.71–1.04]).

**TABLE 2:** Association between patient characteristics and whether a patient's stage at presentation was more advanced than the stage at diagnosis among non‐analytic cases at Moffitt Cancer Center (MCC) diagnosed with breast cancer, colon cancer, lung cancer, melanoma, and prostate cancer between 2008 and 2020.

Baseline characteristics	Match	Stage at presentation more advanced	Odds ratio (95% confidence interval)	
			Cohort‐adjusted^a^	Multivariable
Age				
18–64	3304 (51.5)	1056 (58.6)	1.00 (reference)	1.00 (reference)
65 or higher	3110 (48.5)	746 (41.4)	0.85 (0.76–0.95)	1.03 (0.52–1.85)
Age				
18–39	244 (3.8)	117 (6.5)	1.00 (reference)	Not included^b^
40–64	3060 (47.7)	939 (52.1)	0.86 (0.68–1.09)	
65 or higher	3110 (48.5)	746 (41.4)	0.74 (0.58–0.94)	
Gender				
Female	2892 (45.1)	920 (51.1)	1.00 (reference)	Not included
Male	3521 (54.9)	882 (48.9)	1.09 (0.94–1.25)	
Race				
White	5650 (88.4)	1560 (87)	1.00 (reference)	Not included
Asian	87 (1.4)	32 (1.8)	1.29 (0.84–1.94)	
Black	466 (7.3)	145 (8.1)	1.13 (0.92–1.38)	
Other/multiple	188 (2.9)	57 (3.2)	1.10 (0.80–1.48)	
Ethnicity				
Non‐Hispanic	5947 (93.2)	1635 (91.3)	1.00 (reference)	Dropped due to not significant
Hispanic or Spanish origin	432 (6.8)	156 (8.7)	1.28 (1.05–1.55)	
Payment method (all ages)				
Private/Manage	1332 (37.0)	143 (34.0)	1.00 (reference)	1.00 (reference)
Self‐pay/not insured	119 (3.3)	20 (4.8)	1.49 (0.87–2.44)	1.47 (0.83–2.46)
Medicaid	159 (4.4)	17 (4.0)	1.03 (0.58–1.72)	1.00 (0.55–1.68)
Medicare	1887 (52.4)	232 (55.1)	1.20 (0.96–1.51)	2.17 (1.50–3.11)
Tricare/VA	104 (2.9)	9 (2.1)	0.91 (0.42–1.75)	0.97 (0.36–2.14)
Payment method (age 18–64)				
Private/Manage	1214 (68.7)	131 (59.5)	1.00 (reference)	Reference for interaction term
Self‐pay/not insured	109 (6.2)	18 (8.2)	1.47 (0.83–2.46)	
Medicaid	155 (8.8)	16 (7.3)	0.97 (0.54–1.64)	
Medicare	228 (12.9)	49 (22.3)	2.17 (1.49–3.11)	
Tricare/VA	61 (3.5)	6 (2.7)	0.98 (0.37–2.15)	
Payment method (age 65 and above)				
Private/Manage	118 (6.4)	12 (6.0)	1.00 (reference)	1.00 (reference)
Self‐pay/not insured	10 (0.5)	2 (1.0)	1.72 (0.24–7.85)	1.17 (0.15–5.89)
Medicaid	4 (0.2)	1 (0.5)	3.21 (0.16–24.29)	3.02 (0.14–24.76)
Medicare	1659 (90.5)	183 (91.0)	1.04 (0.58–2.03)	0.48 (0.24–1.02)
Tricare/VA	43 (2.3)	3 (1.5)	0.78 (0.17–2.65)	0.81 (0.15–3.81)
Cigarettes use				
Never	2337 (40.1)	734 (44.5)	1.00 (reference)	Not included
Former	2754 (47.3)	750 (45.4)	1.09 (0.96–1.23)	
Current	730 (12.5)	167 (10.1)	0.90 (0.74–1.09)	
Whether address at diagnosis inside MCC catchment area (15 counties in FL^c^)				
No	1321 (33.3)	215 (40.3)	1.00 (reference)	Dropped due to not significant
Yes	2644 (66.7)	319 (59.7)	0.86 (0.71–1.04)	
Cohort				
Breast	1185 (18.5)	506 (28.1)	Not applicable	1.00 (reference)
Colon	857 (13.4)	273 (15.1)		1.66 (1.18–2.33)
Lung	2188 (34.1)	400 (22.2)		0.77 (0.57–1.05)
Melanoma	676 (10.5)	353 (19.6)		2.40 (1.74–3.33)
Prostate	1508 (23.5)	270 (15.0)		0.57 (0.39–0.83)

^a^ The estimated odds ratio and confidence interval were calculated using logistic regression adjusting for cancer types the patient diagnosed with.

^b^ The three‐group age variable was not included in the multivariable analysis due to it not being compatible with the binary age categorization used in the analysis of interaction between age and payment method.

^c^ The 15‐county MCC catchment area included the following counties in Florida: Charlotte, Citrus, Desoto, Hardee, Hernando, Highlands, Hillsborough, Lake, Lee, Manatee, Pasco, Pinellas, Polk, Sarasota, or Sumter.

Subsequently, patient characteristics that were statistically significant in the cohort‐adjusted analysis were included in a multivariable logistic regression model (Table 2). All factors with the exception of ethnicity and 15‐county catchment area were retained indicating that these factors were associated with a more advanced stage at presentation, while controlling for other characteristics.

No obvious patterns were observed across cancer types after stratification by cancer (Table 3). Certain characteristics remained significantly associated with having a more advanced stage at presentation including age, Medicare insurance, and former smokers.

**TABLE 3:** Association between patient characteristics and whether a patient's stage at presentation was more advanced than the stage at diagnosis among non‐analytic cancer patients at Moffitt Cancer Center (MCC) diagnosed between 2008 and 2020, by cancer type.

Baseline characteristics	Odds Ratio (95% confidence interval)				
	Breast cohort	Lung cohort	melanoma cohort	Colon cohort	Prostate cohort
Age					
18–64	1.00 (Reference)	1.00 (Reference)	1.00 (Reference)	1.00 (Reference)	1.00 (Reference)
65 or higher	0.70 (0.55–0.90)^a^	0.87 (0.70–1.08)	0.71 (0.55–0.92)	1.14 (0.87–1.51)	0.94 (0.72–1.22)
Age					
18–39	1.00 (Reference)	1.00 (Reference)	1.00 (Reference)	1.00 (Reference)	NA
40–64	0.75 (0.53–1.05)	2.01 (0.71–8.43)	0.82 (0.51–1.34)	1.17 (0.67–2.16)	1.00 (reference)
65 or higher	0.55 (0.38–0.80)	1.72 (0.61–7.22)	0.60 (0.38–0.97)	1.32 (0.75–2.45)	0.94 (0.72–1.22)
Gender					
Female	1.00 (Reference)	1.00 (Reference)	1.00 (Reference)	1.00 (Reference)	1.00 (Reference)
Male	0.62 (0.18–1.72)	1.11 (0.89–1.37)	1.01 (0.76–1.34)	1.18 (0.90–1.55)	NA
Race					
White	1.00 (Reference)	1.00 (Reference)	1.00 (Reference)	1.00 (Reference)	1.00 (Reference)
Asian	1.16 (0.59–2.18)	2.02 (0.96–3.96)	0.96 (0.04–10.01)	0.17 (0.01–0.83)	4.08 (1.20–12.89)
Black	1.39 (1.01–1.89)^a^	0.97 (0.54–1.62)	0.32 (0.02–1.87)	0.96 (0.61–1.47)	1.08 (0.69–1.63)
Other/multiple	1.49 (0.91–2.39)	0.74 (0.28–1.61)	1.53 (0.38–5.81)	0.90 (0.41–1.78)	0.90 (0.41–1.76)
Ethnicity					
Non‐Hispanic	1.00 (Reference)	1.00 (Reference)	1.00 (Reference)	1.00 (Reference)	1.00 (Reference)
Hispanic or Spanish origin	1.59 (1.16–2.18)	0.88 (0.54–1.37)	0.96 (0.44–1.95)	1.44 (0.91–2.23)	1.19 (0.69–1.94)
Payment method (all ages)					
Private/Manage	1.00 (Reference)	1.00 (Reference)	1.00 (Reference)	1.00 (Reference)	1.00 (Reference)
Self‐pay/not insured	1.80 (0.59–4.58)	1.07 (0.31–2.84)	0.91 (0.20–2.99)	1.81 (0.61–4.73)	3.14 (0.46–13.18)
Medicaid	1.39 (0.54–3.09)	0.46 (0.11–1.32)	2.84 (0.36–17.80)	1.13 (0.36–3.01)	NA
Medicare	1.28 (0.78–2.11)	0.89 (0.59–1.37)	1.17 (0.73–1.89)	1.30 (0.76–2.27)	2.09 (1.04–4.56)
Tricare/VA	NA	0.56 (0.09–1.94)	1.60 (0.34–5.86)	0.90 (0.05–5.41)	2.76 (0.59–9.64)
Payment method (age 18–64)					
Private/Manage	1.00 (reference)	1.00 (reference)	1.00 (reference)	1.00 (reference)	1.00 (reference)
Self pay/Not insured	1.47 (0.42–4.00)	1.31 (0.37–3.57)	0.67 (0.10–2.61)	1.87 (0.63–4.96)	2.99 (0.44–12.61)
Medicaid	1.23 (0.45–2.87)	0.49 (0.11–1.43)	2.92 (0.37–18.37)	1.06 (0.33–2.83)	NA
Medicare	3.58 (1.78–6.96)^a^	1.50 (0.71–2.99)	2.05 (0.73–5.30)	1.66 (0.67–3.84)	2.76 (0.90–7.77)
Tricare/VA	NA	1.35 (0.21–5.04)	1.25 (0.18–5.49)	NA	2.99 (0.44–12.61)
Payment method (age 65 and above)					
Private/Manage	1.00 (Reference)	1.00 (Reference)	1.00 (Reference)	1.00 (Reference)	1.00 (Reference)
Self pay/Not insured	NA	NA	3.00 (0.10–94.48)	NA	NA
Medicaid	7.00 (0.22–239.93)	NA	NA	NA	NA
Medicare	0.56 (0.14–3.74)	0.68 (0.30–1.82)	0.78 (0.22–3.59)	3.04 (0.59–55.91)	NA
Tricare/VA	NA	NA	3.00 (0.10–94.48)	5.33 (0.18–164.46)	NA
Cigarettes use					
Never	1.00 (Reference)	1.00 (Reference)	1.00 (Reference)	1.00 (Reference)	1.00 (Reference)
Former	1.17 (0.93–1.48)	1.54 (1.11–2.19)	0.88 (0.66–1.18)	0.99 (0.73–1.35)	0.93 (0.71–1.23)
Current	1.28 (0.87–1.85)	1.01 (0.66–1.55)	0.94 (0.57–1.52)	0.82 (0.49–1.31)	0.68 (0.38–1.13)
Whether address at diagnosis inside MCC catchment area (15 counties in FL^b^)					
No	1.00 (reference)	1.00 (reference)	1.00 (reference)	1.00 (reference)	1.00 (reference)
Yes	0.78 (0.52–1.19)	0.99 (0.68–1.44)	0.75 (0.50–1.11)	1.00 (0.63–1.61)	0.79 (0.45–1.43)

^a^ Factors remained significant in a multivariable logistic regression with backward elimination process.

^b^ The 15‐county MCC catchment area included the following counties in Florida: Charlotte, Citrus, Desoto, Hardee, Hernando, Highlands, Hillsborough, Lake, Lee, Manatee, Pasco, Pinellas, Polk, Sarasota, or Sumter.

### 3.3 Length of time between date of diagnosis and date of presentation

Among non‐analytic cases, we examined the time gap between date of diagnosis and date of presentation at MCC in association with patient characteristics (Table 4). Patients aged ≥65 (median = 296 days) had a significantly shorter gap in the two timepoints as compared with those aged 18–64 (median = 455 days). Male patients (median = 400 days) showed a longer lag time as compared with female patients (median = 343 days). Differences were observed by race, with shorter gaps observed among Asians (median = 347 days) and Whites (median = 368 days) as compared to Blacks (median = 510 days) and those of other race or multiple races (median = 475 days). Patients aged <65 with Medicare (median = 337 days) had a longer time gap between diagnosis and presentation as compared with patients using other payment methods (median = 76–100 days). Ever smokers (median = 308–348 days) presented at MCC sooner as compared with never smokers (median = 433 days). Additionally, patients residing in the MCC 15‐county catchment area (median = 80 days) had a shorter time gap as compared with those residing outside the catchment area (median = 151–204 days, Table 4). Lung cancer patients (median = 199 days) had the shortest lag time compared with patients diagnosed with other cancers.

**TABLE 4:** Association between patient characteristics and time between date of diagnosis and date of presentation at Moffitt Cancer Center (MCC) among non‐analytic patients.

Baseline characteristics	Days from cancer diagnosis to presentation (among non‐analytic cases)	
	Median (IQR)	*p*‐value^a^
Age		
18–64	454.5 (930.8)	<0.001
65 or higher	296 (716.0)	
Age		
18–39	444.5 (794.2)	<0.001
40–64	455 (946.5)	
65 or higher	296 (716)	
Gender		
Female	343 (815.8)	<0.001
Male	400 (856.2)	
Race		
White	368 (824.8)	<0.001
Asian	346.5 (935.5)	
Black	510 (861.8)	
Other/multiple	475 (942)	
Ethnicity		
Non‐Hispanic	377 (832.0)	0.136
Hispanic or Spanish origin	403 (838)	
Payment method (all ages)		
Private/Manage	72 (319.8)	0.861
Self pay/Not insured	85 (252)	
Medicaid	100 (253)	
Medicare	74.5 (336.0)	
Tricare/VA	80.5 (333)	
Payment method (age 18–64)		
Private/Manage	76 (328.0)	<0.001
Self pay/Not insured	95.5 (253.5)	
Medicaid	100 (249)	
Medicare	336.5 (906.2)	
Tricare/VA	99 (388)	
Payment method (age 65 and above)		
Private/Manage	52 (185.5)	0.52
Self pay/not insured	19 (167.5)	
Medicaid	29 (160.8)	
Medicare	60.5 (268)	
Tricare/VA	63 (71)	
Cigarettes use		
Never	433 (931.0)	<0.001
Former	348 (819.0)	
Current	308 (764.5)	
Whether address at diagnosis inside MCC catchment area (15 counties in FL^b^)		
No	151 (501.5)	<0.001
Yes	80 (364.5)	
Cohort		
Breast	488.5 (1056.8)	<0.001
Lung	199 (465.5)	
Melanoma	442 (844)	
Colon	436 (711.2)	
Prostate	575.5 (1217.5)	

^a^ *p*‐values were calculated using Wilcoxon rank‐sum test for binary factors and Kruskal–Wallis test for factors with more than two categories.

^b^ The 15‐county MCC catchment area included the following counties in Florida: Charlotte, Citrus, Desoto, Hardee, Hernando, Highlands, Hillsborough, Lake, Lee, Manatee, Pasco, Pinellas, Polk, Sarasota, or Sumter.

Time between cancer diagnosis and the initiation of first course treatment was significantly longer among Black patients (median = 16.5 days) as compared to White patients (median = 9.5 days, *p*‐value = 0.0003). No statistically significant differences were observed between White and Asian or other/multiple races or between Hispanic and non‐Hispanic patients. Longer time intervals were observed between the end of first course treatment and presentation at MCC among racial minorities (Asian [median = 642 days], Black [median = 555.5 days], and other/multiple races [608 days]) as compared with White patients (median = 516.5 days). However, this result did not reach statistical significance (*p*‐value = 0.095). The time between end of first course treatment and presentation to Moffitt was significantly longer among Hispanic patients (median = 604 days) compared to non‐Hispanic patients (median = 519 days, *p*‐value = 0.030).

**FIGURE 2:**
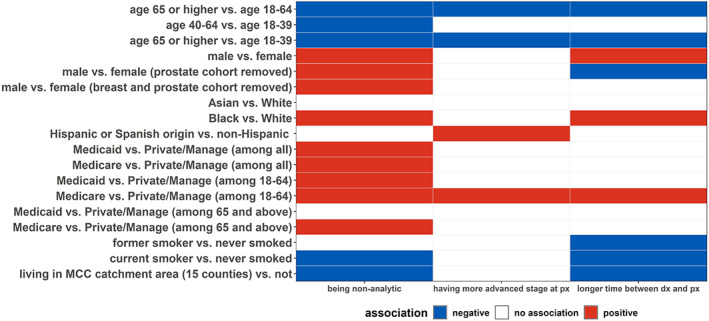
Patients with older age were less likely to be non‐analytic, less likely to have a more advanced stage at presentation, and less likely to have a longer time between presentation at MCC and diagnosis, as compared with younger patients. A similar pattern was observed for patients living outside of the MCC catchment area as compared with those who lived within the catchment area. On the other hand, patients between age 18–64 with Medicare were more likely to be non‐analytic, more likely to have a more advanced stage at presentation, and more likely to have a longer time between date of diagnosis and presentation at MCC. Male patients, as compared with female patients, were more likely to be non‐analytic cases with longer time between date of diagnosis and presentation at MCC. Similar associations were observed for Black patients as compared with White patients.

In summary, patients aged ≥65 were associated with being an analytic case, having stage concordant at presentation, and a shorter time between diagnosis and presentation (Figure 2). Males were more likely to be non‐analytic cases and had longer time between diagnosis and presentation as compared to females, with a similar pattern observed for Black patients as compared with White patients (Figure 2). Medicare users, particularly among those aged 18–64, were more likely to be non‐analytic cases, to have more advanced stage at presentation, and to have longer time gap between diagnosis and presentation as compared with patients with private/managed insurance. Current smokers, as compared with those who never smoked, were more likely to be analytic cases with shorter gap in time between diagnosis and presentation. Generally, patients who lived in the MCC catchment area were associated with being an analytic case (Figure 2).

## 4 DISCUSSION

Cancer care has become increasingly complex over the last several years. Inherent to optimal cancer treatment is multidisciplinary care which can be difficult to institutionalize outside of an NCICC. Ensuring accurate pathologic diagnosis, selection of optimal first‐line therapies based on precision medicine findings, and access to novel clinical trials are all important in modern‐day cancer care. Seamless connectivity between all these facets of care is obligatory to providing the best care and mitigating disparities. To our knowledge, this is the first use of cancer registry data to analyze referral timing differences among different demographic groups to an NCICC.

To this end, our data demonstrates a few notable results. First, our catchment area population was more likely to be analytic cases compared to the population residing outside of the catchment area. This is not surprising since prior data suggests travel time to a NCICC is one of the most influential determinants to receipt of care.^13^ In one study that surveyed a nationally representative cohort of panel members recruited through the GfK method^14^ (*n* = 1016), most individuals stated they would be willing to travel significant amounts to access high‐volume or high‐quality cancer care. However, when participants were asked to identify specific logistical barriers that would prevent them from traveling 1‐hour to access high‐quality cancer care, participants cited barriers such as parking costs, drive time, and cost of lodging.^15^ Although our data does not ascertain etiology, distance along with physician referral patterns and the capacity of a specific area to care for a patient's complex cancer case likely contribute to this difference.

Interestingly compared to non‐Hispanic White patients, Black patients were more likely to be non‐analytic cases and have the longest time interval between diagnosis and presentation to MCC. Among non‐analytic cases, Hispanic patients presented with more advanced stage compared to non‐Hispanic patients and a longer time interval between end of first course treatment and presentation to MCC. While our study could not determine the underlying reasons for these differences, it is possible that structural barriers including but not limited to lack of access to reliable transportation, insurance coverage, and other socioeconomic or geographic factors contributed to our findings. While to our knowledge no previous studies have examined racial and ethnic differences in the timing of receipt of care to an NCICC, studies have analyzed general patterns by different demographic groups, though the results are somewhat mixed. One study of the California Cancer Registry found no difference in utilization of NCICCs between Black and White patients, though there was an inverse association between Hispanic ethnicity and use of an NCICC.^16^ Another study using the SEER database found improvements in overall survival for White patients with myeloma who received care at an NCICC, but these improvements were not observed among Black patients.^17^ Studying how quickly patients receive care at a center is critical to mitigating disparities in outcomes. Certain factors including advanced stage at presentation to an NCICC appear to be correlated with timing of care, which is a common surrogate of delayed referrals or other factors obstructing receipt of care. This is critically important as existing data suggests that certain minority groups initially present at a later stage compared to White patients.^18^ Regarding the observed racial disparities, expansive initiatives dedicated to increasing satellite clinics and telehealth options which would establish a stronger link to an NCICC. Some data suggests that satellite clinics can increase healthcare access for different racial/ethnic groups including Native Americans and Asians, although less of a benefit was seen for Hispanic and Black patients.^19^ Increased telehealth visits at one NCICC resulted in significant cost‐savings and reduction of financial toxicity for patients seeking cancer care.^20^ As these approaches to care delivery become a more routine part of cancer care, it will be important to evaluate the impact on access to guideline‐based care or clinical trials for populations that have been less likely to access care at NCICCs (e.g., racial and ethnic minorities, those living further away from NCICCs).

Additionally, Medicare and Medicaid patients were more likely to be non‐analytic cases compared to patients with private/managed insurance, possibly reflecting delays in insurance approvals of referrals. Of note, access to NCICC may be limited by federal marketplace plans. Only 41% of provider networks initially available on the federal marketplace through the Affordable Care Act included a NCICC.^21^ These restrictions may be particularly challenging for patients requiring complex coordination of cancer care that is generally more readily available at an NCICC.^22^ Given the potential differences in outcome between patients who access an NCICC earlier in their disease course than others, this may represent an opportunity to accelerate insurance approvals.

Interestingly, males were more likely to be non‐analytic cases as compared with females in our study. Men are known to participate less in recommended cancer screenings,^23^ and since our study focused primarily on screen‐detectable cancers, the fact that men were more likely to present to an NCICC later in their diagnosis is not unexpected.

Cancer registry data has many strengthens as it employs uniform case ascertainment, documented stage at presentation and at diagnosis. However, we acknowledge several limitations. First, our analysis was limited to Moffitt Cancer Center, and our findings may not be generalizable to other NCICC's. However, other NCICC's can replicate our analysis using their own Cancer Registry data to examine demographic factors associated with receipt of care at their institutions, highlighting potential disparities most important to address locally. Second, cancer registry data does not capture information on referral source or second opinion visits. Future analyses are planned to examine referral patterns using multiple institutional data sources beyond the Cancer Registry. Our analysis of social determinants of health was limited to insurance status, while other important variables such as transportation costs, childcare, or other issues that may make it difficult for a patient to receive care an NCICC were not available. Future studies will seek to elucidate the factors contributing to the observed differences in timing of receipt of care at an NCICC.

In summary, our study represents an innovative use of cancer registry data to analyze differences in timing of receipt of care at an NCICC. The use of cancer registry data can be leveraged for future studies assessing whether timing differences in referral to an NCICC correlate with long‐term outcomes will be important in understanding the translational impact of these observed disparities. Overall, our study underscores the utility of using cancer registry data to better understand disparities in timing of access to cancer care at specialized centers and may help inform targeted interventions to improve access in the future.

## AUTHOR CONTRIBUTIONS

**Kedar Kirtane:** Conceptualization (equal); funding acquisition (equal); writing – original draft (equal). **Yayi Zhao:** Conceptualization (equal); data curation (equal); formal analysis (equal); methodology (equal); writing – original draft (equal). **Rossybelle P Amorrortu:** Conceptualization (equal); project administration (equal); writing – review and editing (equal). **Lindsay Fuzzell:** Conceptualization (equal); writing – review and editing (equal). **Susan Vadaparampil:** Conceptualization (equal); writing – review and editing (equal). **Dana Rollison:** Conceptualization (equal); methodology (equal); project administration (equal); supervision (equal); writing – review and editing (equal).

## FUNDING INFORMATION

This work has been supported by a Miles for Moffitt Grant and the Collaborative Data Services Core at the H. Lee Moffitt Cancer Center & Research Institute, a comprehensive cancer center designated by the National Cancer Institute and funded in part by Moffitt's Cancer Center Support Grant (P30‐CA076292).

## CONFLICT OF INTEREST STATEMENT

Rossybelle Amorrortu, Lindsay Fuzzell, Yayi Zhao, and Susan Vadaparampil declare they have no conflicts of interest. Kedar Kirtane owns stock in Seattle Genetics, Oncternal Therapeutics, and Veru. Dana Rollison serves on the Board of Directors for NanoString Technologies, Inc.

## Data Availability

The data presented in this article are not readily available to protect participant confidentiality and privacy. Direct requests to access the datasets to the corresponding author.

## APPENDIX A Distribution of stage at presentation and stage at diagnosis for Moffitt Cancer Center patients diagnosed with breast cancer, colon cancer, lung cancer, melanoma, and prostate cancer between 2008 and 2020, presented by cancer type and analytic status.

### A.1

A similar pattern was observed for the distribution of stage at presentation and stage at diagnosis when stratifying the previous analysis by cancer type. The majority of analytic patients were found to have a concordant stage at presentation and at diagnosis. The proportions of non‐analytic patients whose stage at presentation was more advanced than their stage at diagnosis varied by cancer site (breast = 28%, colon = 22.5%, lung = 14.5%, melanoma = 29.2%, prostate = 13.8%).

## References

[1] NCI‐Designated Cancer Centers: National Cancer Insitute; [updated June 24, 2019 April 07, 2023]. Available from: https://www.cancer.gov/research/infrastructure/cancer‐centers

[2] Birkmeyer NJ , Goodney PP , Stukel TA , Hillner BE , Birkmeyer JD . Do cancer centers designated by the National Cancer Institute have better surgical outcomes? Cancer. 2005;103(3):435‐441. 15622523 10.1002/cncr.20785

[3] Wolfson JA , Sun CL , Wyatt LP , Hurria A , Bhatia S . Impact of care at comprehensive cancer centers on outcome: results from a population‐based study. Cancer. 2015;121(21):3885‐3893. 26218755 10.1002/cncr.29576 PMC4892698

[4] McDaniels‐Davidson C , Feng CH , Martinez ME , et al. Improved survival in cervical cancer patients receiving care at National Cancer Institute‐designated cancer centers. Cancer. 2022;128(19):3479‐3486. doi:10.1002/cncr.34404. Epub 20220802. PubMed PMID: 35917201. 35917201 PMC9544648

[5] Murimwa GZ , Karalis JD , Meier J , et al. ASO Visual Abstract: Hospital designations and their impact on guideline‐concordant care and survival in pancreatic cancer: Do they matter? Ann Surg Oncol. 2023. doi:10.1245/s10434-023-13452-0 36964844

[6] Paulson EC , Mitra N , Sonnad S , et al. National Cancer Institute designation predicts improved outcomes in colorectal cancer surgery. Ann Surg. 2008;248(4):675‐686. 18936581 10.1097/SLA.0b013e318187a757

[7] Roghmann F , Ravi P , Hanske J , et al. Perioperative outcomes after radical cystectomy at NCI‐designated centres: are they any better? Can Urol Assoc J. 2015;9(5–6):207‐212. 26225174 10.5489/cuaj.2621 PMC4479646

[8] Ho G , Wun T , Muffly L , et al. Decreased early mortality associated with the treatment of acute myeloid leukemia at National Cancer Institute‐designated cancer centers in California. Cancer. 2018;124(9):1938‐1945. Epub 20180216. doi:10.1002/cncr.31296 PubMed PMID: 29451695; PMCID: PMC6911353. 29451695 PMC6911353

[9] Shulman LN , Palis BE , McCabe R , et al. Survival as a quality metric of cancer care: use of the National Cancer data base to assess hospital performance. J Oncol Pract. 2018;14(1):e59‐e72. Epub 20171101. doi:10.1200/jop.2016.020446 PubMed PMID: 29091535. 29091535

[10] Bristow RE , Chang J , Ziogas A , Campos B , Chavez LR , Anton‐Culver H . Impact of National Cancer Institute comprehensive cancer centers on ovarian cancer treatment and survival. J Am Coll Surg. 2015;220(5):940‐950. Epub 20150214. doi:10.1016/j.jamcollsurg.2015.01.056 PubMed PMID: 25840536; PMCID: PMC5145798. 25840536 PMC5145798

[11] Polite BN , Adams‐Campbell LL , Brawley OW , et al. Charting the future of cancer health disparities research: a position statement from the American Association for Cancer Research, the American Cancer Society, the American Society of Clinical Oncology, and the National Cancer Institute. Cancer Res. 2017;77(17):4548‐4555. 28739629 10.1158/0008-5472.CAN-17-0623

[12] Khorana AA , Tullio K , Elson P , et al. Time to initial cancer treatment in the United States and association with survival over time: an observational study. PLoS ONE. 2019;14(3):e0213209. 30822350 10.1371/journal.pone.0213209 PMC6396925

[13] Onega T , Duell EJ , Shi X , Demidenko E , Goodman D . Determinants of NCI Cancer Center attendance in Medicare patients with lung, breast, colorectal, or prostate cancer. J Gen Intern Med. 2009;24(2):205‐210. 19067086 10.1007/s11606-008-0863-y PMC2628988

[14] Knowledge Panel (United States): GfK Global; [cited 2023 April 07, 2023]. Available from: http://www.gfk.com/products‐a‐z/us/knowledgepanel‐united‐states/

[15] Resio BJ , Chiu AS , Hoag JR , et al. Motivators, barriers, and facilitators to traveling to the safest hospitals in the United States for complex cancer surgery. JAMA Netw Open. 2018;1(7):e184595‐e. 30646367 10.1001/jamanetworkopen.2018.4595 PMC6324377

[16] Huang LC , Ma Y , Ngo JV , Rhoads KF . What factors influence minority use of National Cancer Institute‐designated cancer centers? Cancer. 2014;120(3):399‐407. 24452674 10.1002/cncr.28413 PMC3905240

[17] Ailawadhi S , Advani P , Yang D , et al. Impact of access to NCI‐and NCCN‐designated cancer centers on outcomes for multiple myeloma patients: a SEER registry analysis. Cancer. 2016;122(4):618‐625. 26565660 10.1002/cncr.29771

[18] Miller KD , Goding Sauer A , Ortiz AP , et al. Cancer statistics for hispanics/latinos, 2018. CA Cancer J Clin. 2018;68(6):425‐445. 30285281 10.3322/caac.21494

[19] Onega T , Alford‐Teaster J , Wang F . Population‐based geographic access to parent and satellite National Cancer Institute Cancer Center Facilities. Cancer. 2017;123(17):3305‐3311. 28464212 10.1002/cncr.30727

[20] Patel KB , Turner K , Tabriz AA , et al. Estimated indirect cost savings of using telehealth among nonelderly patients with cancer. JAMA Netw Open. 2023;6(1):e2250211‐e. 36626174 10.1001/jamanetworkopen.2022.50211 PMC9856804

[21] Kehl KL , Keating NL , Giordano SH , Schrag D . Insurance networks and access to affordable cancer care. J Clin Oncol. 2020;38(4):310‐315. 31804867 10.1200/JCO.19.01484 PMC6994255

[22] Ver Hoeve ES , Simon MA , Danner SM , et al. Implementing patient navigation programs: considerations and lessons learned from the Alliance to advance patient‐centered cancer care. Cancer. 2022;128(14):2806‐2816. Epub 20220517. doi:10.1002/cncr.34251 PubMed PMID: 35579501; PMCID: PMC9261966. 35579501 PMC9261966

[23] Davis JL , Buchanan KL , Katz RV , Green BL . Gender differences in cancer screening beliefs, behaviors, and willingness to participate: implications for health promotion. Am J Mens Health. 2012;6(3):211‐217. 22071507 10.1177/1557988311425853 PMC3776317

